# Inorganic Phosphate-Induced Extracellular Vesicles from Vascular Smooth Muscle Cells Contain Elevated Levels of Hyaluronic Acid, Which Enhance Their Interaction with Very Small Superparamagnetic Iron Oxide Particles

**DOI:** 10.3390/ijms25052571

**Published:** 2024-02-22

**Authors:** Christian Freise, Karina Biskup, Véronique Blanchard, Jörg Schnorr, Matthias Taupitz

**Affiliations:** 1 Department of Radiology, Campus Mitte, Charité—Universitätsmedizin Berlin, Corporate Member of Freie Universität Berlin and Humboldt-Universität zu Berlin, Charitéplatz 1, 10117 Berlin, Germany; joerg.schnorr@charite.de (J.S.); matthias.taupitz@charite.de (M.T.); 2 Institute of Diagnostic Laboratory Medicine, Clinical Chemistry and Pathobiochemistry, Campus Virchow-Klinikum, Charité—Universitätsmedizin Berlin, Corporate Member of Freie Universität Berlin and Humboldt-Universität zu Berlin, Augustenburger Platz 1, 13353 Berlin, Germany; karina.biskup@medicalschool-berlin.de (K.B.); veronique.blanchard@medicalschool-berlin.de (V.B.); 3 Department of Human Medicine, Medical School Berlin, Rüdesheimer Str. 50, 14197 Berlin, Germany

**Keywords:** extracellular vesicles, inorganic phosphate, uremic toxins, VSOP, hyaluronic acid, glycosaminoglycans, exosomes

## Abstract

Patients with chronic kidney disease (CKD) have a high prevalence of hyperphosphatemia, where uremic toxins like inorganic phosphate (Pi) induce a cardiovascular remodeling. Related disorders like atherosclerosis bear the risk of increased morbidity and mortality. We previously found that Pi stimulates the synthesis and sulfation of the negatively charged glycosaminoglycans (GAGs) heparan sulfate and chondroitin sulfate in vascular smooth muscle cells (VSMC). Similar GAG alterations were detected in VSMC-derived exosome-like extracellular vesicles (EV). These EV showed a strong interaction with very small superparamagnetic iron oxide particles (VSOP), which are used as imaging probes for experimental magnetic resonance imaging (MRI). Hyaluronic acid (HA) represents another negatively charged GAG which is supposed to function as binding motif for VSOP as well. We investigated the effects of Pi on the amounts of HA in cells and EV and studied the HA-dependent interaction between VSOP with cells and EV. Rat VSMC were treated with elevated concentrations of Pi. CKD in rats was induced by adenine feeding. EV were isolated from culture supernatants and rat plasma. We investigated the role of HA in binding VSOP to cells and EV via cell-binding studies, proton relaxometry, and analysis of cellular signaling, genes, proteins, and HA contents. Due to elevated HA contents, VSMC and EV showed an increased interaction with VSOP after Pi stimulation. Amongst others, Pi induced hyaluronan synthase (HAS)2 expression and activation of the Wnt pathway in VSMC. An alternative upregulation of HA by iloprost and an siRNA-mediated knockdown of HAS2 confirmed the importance of HA in cells and EV for VSOP binding. The in vitro-derived data were validated by analyses of plasma-derived EV from uremic rats. In conclusion, the inorganic uremic toxin Pi induces HA synthesis in cells and EV, which leads to an increased interaction with VSOP. HA might therefore be a potential molecular target structure for improved detection of pathologic tissue changes secondary to CKD like atherosclerosis or cardiomyopathy using EV, VSOP and MRI.

## 1. Introduction

A decline in kidney function leads to the accumulation of uremic toxins such as organic indoxyl sulfate and p-cresyl sulfate or inorganic phosphate (Pi). They play a crucial role in the progression of chronic kidney disease (CKD) and could exert massive pathophysiological effects on different organ systems [1,2]. For instance, elevated concentrations of Pi, i.e., hyperphosphatemia, influence the development of bone and cartilage and are related to diabetes. In addition, elevated concentrations of Pi are also correlated with vascular calcification [3], which entails a high risk of cardiovascular events and mortality [4]. This includes effects of Pi on the remodeling of the extracellular matrix (ECM), e.g., by vascular smooth muscle cells (VSMC) and in extracellular vesicles (EV) [2,5].

EV such as exosomes or microvesicles are released by multiple types of cells, tissues, and organs, and are known to function as paracrine mediators of intercellular communication including pathophysiological processes such as the binding of tumor cell-derived fibronectin-containing microvesicles to non-transformed fibroblasts. This enables adhesion-independent growth, which favors the generation of tumors [6,7].

The above-mentioned effects of Pi on the remodeling of the surface structure of vascular cells and isolated EV include the induction of higher contents of sulfated glycosaminoglycans (GAGs) such as heparan sulfate and chondroitin sulfate [5]. On cells, these GAGs are supposed to interact with protein ligands such as the cluster of differentiation 44 or glypican-1 via electrostatic interaction, thereby affecting the interaction of EV with cells and physiological functions in all organ systems [8,9]. The role of GAGs on EV for their interaction with ligands such as fibroblast growth factors, transforming growth factors or Wnt proteins, has so far only been investigated in a few studies. We recently found that heparan sulfate and chondroitin sulfate contribute to the interaction of EV with very small superparamagnetic iron oxide particles (VSOP) [5].

Besides heparan sulfate and chondroitin sulfate, hyaluronic acid (HA) represents another central component of cellular surface structures that is abundantly expressed in various organs [10]. HA contains carboxylic groups, which cause a negative charge of HA molecules. It consists of the repeating polysaccharides D-glucuronic acid (GlcUA) and *N*-acetylglucosamine (GlcNAc) and is synthesized at the inner surface of the plasma membrane by hyaluronan synthases (HAS) [11].

HA influences cellular functions and is involved in various diseases. Amongst others, HA promotes VSMC proliferation and migration [12]. In addition, it contributes to the progression and increased disruption of vascular integrity in atherosclerosis [13], which is associated with an overexpression of HAS2 [14]. Further, HA influences proinflammatory processes such as the recruitment of macrophages [15].

Vice versa, various stimuli regulate HA expression. Hyperglycemic conditions in sera or culture media promote HA synthesis in VSMC [16]. Growth factors and cytokines also increase HAS expression and, subsequently, HA synthesis [17].

A common feature of HA and other GAGs is their negative charge due to the abundance of carboxylic groups. In contrast to HA, other GAGs like heparan sulfate and chondroitin sulfate also contain negatively charged sulfate groups [18]. Negatively charged surface structures are of interest for monitoring pathophysiological alterations by magnetic resonance imaging (MRI). Gadolinium-containing contrast agents preferably bind to sulfated dextran molecules compared to non-sulfated dextran [19]. And VSOP are suggested to interact with inflamed tissues by the binding of their cationic iron oxide cores to negatively charged sulfate groups on surfaces [20]. VSOP is an experimental imaging probe which is of interest for MRI-based monitoring of, e.g., multiple sclerosis, atherosclerosis, and inflammatory bowel disease [20,21,22].

We previously demonstrated that Pi-induced EV, with contain high amounts of sulfated GAGs, also strongly interact with VSOP [5]. However, the interaction between VSOP and EV might also occur via other negatively charged surface structures like HA.

A central aim of the present study was therefore to investigate the effects of the inorganic uremic toxin Pi on the contents of non-sulfated HA in cells and EV with subsequent analyses of the consequences of the interaction between them and VSOP.

## 2. Results

To obtain a compact overview of the research process, a corresponding diagram is shown in Supplementary Figure S1.

### 2.1. Pi induces the Synthesis of HA in VSMC and in Their Isolated EV

**Figure 1:**
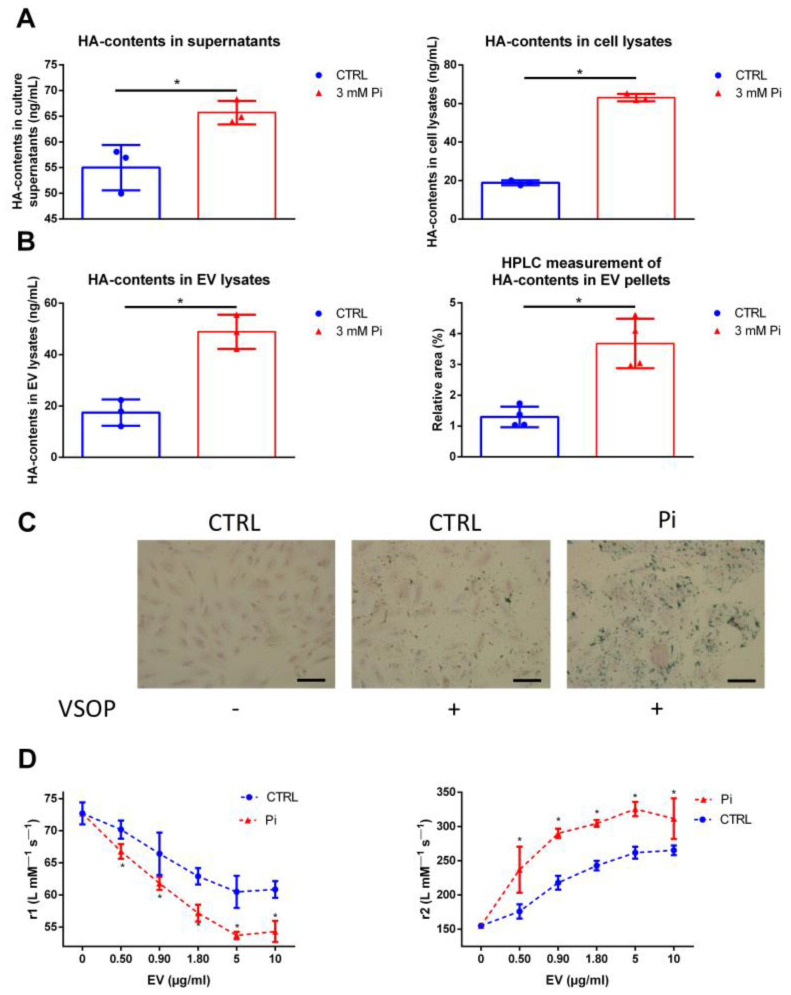
Inorganic phosphate (Pi) induces elevated HA contents in VSMC and isolated EV and increases their interaction with VSOP. (**A**) VSMC were treated with 3 mM Pi for 7 d. HA contents in supernatants and cell lysates were analyzed by ELISA measurements (means ± SD; n = 3; * *p* < 0.05). (**B**) EV were isolated from VSMC culture supernatants. HA contents of EV pellets were analyzed by ELISA and HPLC measurements (means ± SD; n = 4; * *p* < 0.05). (**C**) VSMC were treated with 3 mM Pi or left untreated prior to the incubation with VSOP. After washing the cells, bound VSOP were visualized by iron staining and appeared blue. Shown are representative images out of three independent experiments. Bars = 50 µm. (**D**) VSOP relaxivities (r1, r2) were measured with or without the presence of rising EV concentrations. The r1/r2-values were determined by linear fitting of T1- and T2-relaxation rates in relation to VSOP concentrations (means ± SD; n = 4; * *p* < 0.05). EV, extracellular vesicles; HA, hyaluronic acid; Pi, inorganic phosphate; r1, T1-relaxivity of VSOP; r2, T2-relaxivity of VSOP; VSMC, vascular smooth muscle cells; VSOP, very small superparamagnetic iron oxide nanoparticles.

We initially analyzed whether treatment with the uremic toxin Pi influences the HA contents in VSMC and in secreted EV. ELISA measurements revealed that supernatants and cell lysates of Pi-treated cells contained significantly higher HA contents compared to the respective controls (Figure 1A). In addition, lysates of EV that were isolated from supernatants of Pi-treated cells contained more HA compared to lysates of control EV, as shown by ELISA and HPLC analyses (Figure 1B). We next aimed to study whether higher HA contents in cells and EV affect their interaction with VSOP. For this, VSMC were incubated with VSOP and bound VSOP were visualized by iron staining. Incubation of VSMC control cells with VSOP revealed no relevant binding of VSOP to the control cells (Figure 1C). In contrast, Pi-treated cells with higher HA contents displayed intense positively stained areas around the cells (Figure 1C). We subsequently used ^1^H-NMR-relaxometry measurements to also determine an interaction between VSOP and isolated EV. An interaction would affect the local magnetic field of VSOP and therefore decrease the T1-relaxivities (r1) and increase the T2-relaxivities (r2) of VSOP. Indeed, the Pi-induced EV, which are rich in HA, showed a stronger effect on the decline in the T1-relaxivity and on the increase in the T2-relaxivity of VSOP compared to less HA-containing CTRL vesicles (Figure 1D).

### 2.2. Pi Induces HAS1 and HAS2 in VSMC

**Figure 2:**
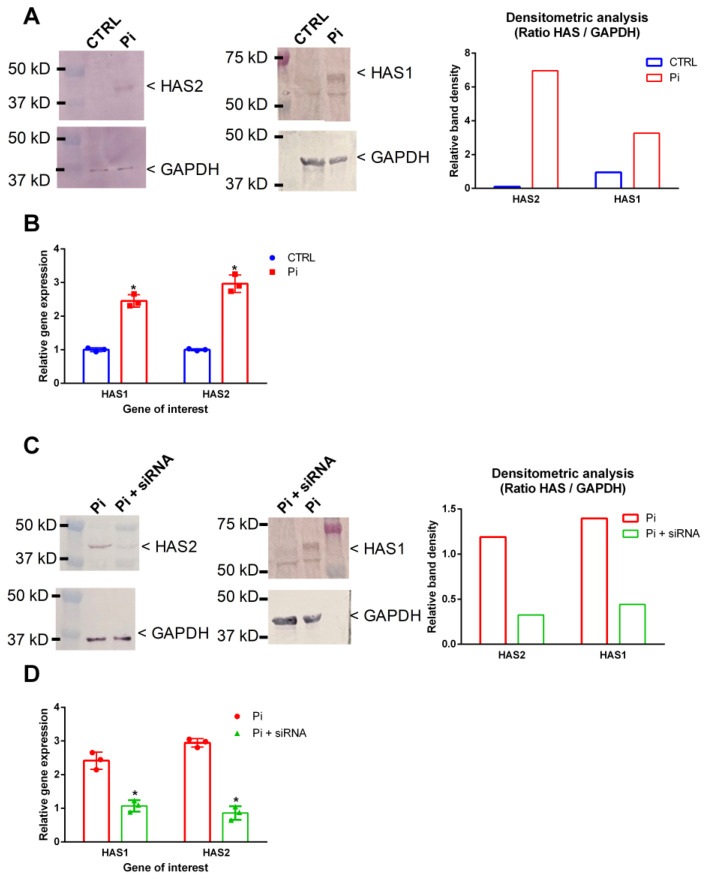
Pi induces HAS1 and HAS2 in VSMC, which can be blocked by specific siRNA. (**A**) VSMC were treated for 7d with Pi or left untreated. HAS1/2 protein expression determined by Western blot. Representative blots along with a densitometric band analysis are shown from three independent experiments. (**B**) VSMC were treated with Pi for 2 d. Mean gene expressions of HAS1/2 (n = 3) were determined by qPCR and normalized to RPL19 expressions. * *p* < 0.05 versus control. (**C**) VSMC were treated with 100 ng HAS1/2 siRNA prior to the treatment with Pi as described above. Effects on protein expression were determined by Western blot. Representative blots along with a densitometric band analysis are shown from three independent experiments. (**D**) VSMC were treated with HAS1/2 siRNA prior to the treatment with Pi as described above. Effects on gene expression were determined by qPCR (n = 3, * *p* < 0.05 compared to control). EV, extracellular vesicles; GAPDH, glyceraldehyde 3-phosphate dehydrogenase; HAS, hyaluronan synthase; Pi, inorganic phosphate; RPL19, ribosomal protein L19; VSMC, vascular smooth muscle cells.

Further Western blot analyses of intracellular effects of Pi showed that treatment of VSMC with Pi led to higher protein expression of HAS1 and of HAS2 compared to that of the controls (Figure 2A). Similar effects were observed on the gene expression levels (Figure 2B). A siRNA-mediated approach distinctly counteracted the Pi-mediated effects on HAS1’s and HAS2’s protein (Figure 2C) and gene expressions (Figure 2D).

### 2.3. Reduced HA Contents in Cells and EV Attenuate Their Interaction with VSOP

**Figure 3:**
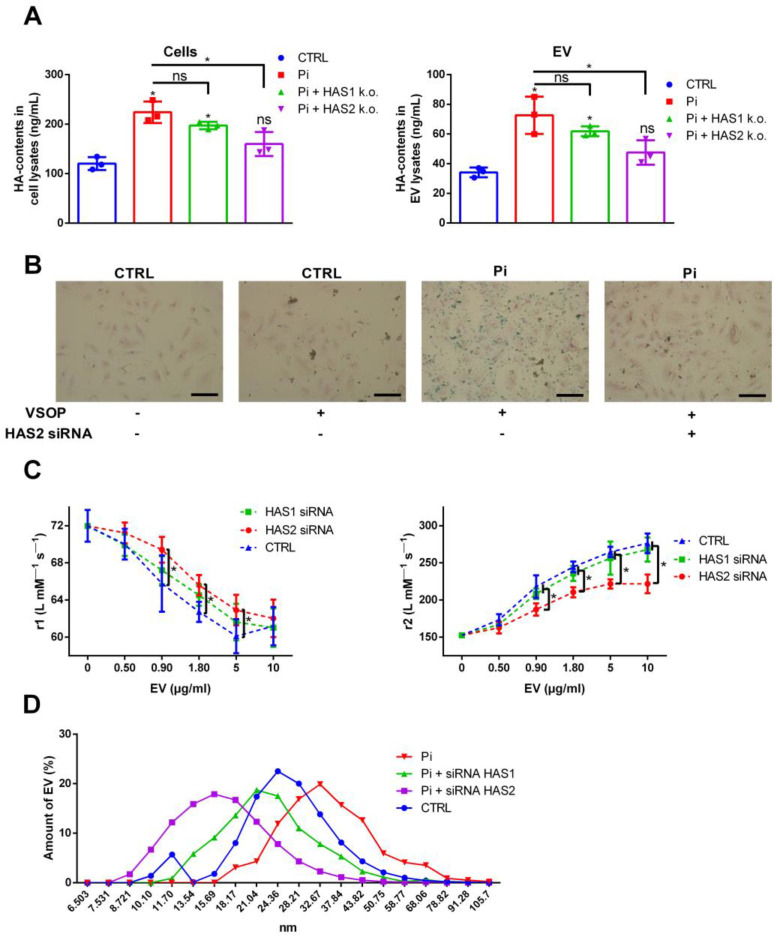
Downregulation of HAS2 blocks the Pi-mediated induction of HA in VSMC and EV and thereby reduces the interaction of EV with VSOP. (**A**) VSMC were treated with HAS1/2 siRNA prior to the treatment with Pi for 7 d. HA contents were measured by ELISA (means ± SD; n = 3; * *p* < 0.05 versus control and as indicated; ns, not significant). (**B**) VSMC were treated as in (**A**). After 7 d, the cells were incubated with VSOP. After washing the cells, bound VSOP were visualized by iron staining and appeared blue. Shown are representative images out of three independent experiments. Bars = 50 µm. (**C**) VSMC were treated as in (**A**). EV were isolated from culture supernatants and were mixed at different concentrations with VSOP. Resulting effects on VSOP relaxivities (r1, r2) were determined by linear fitting of T1- and T2-relaxation rates in relation to VSOP concentrations (means ± SD; n = 4; * *p* < 0.05 as indicated). (**D**) Hydrodynamic diameters of the EVs from (**C**) were determined by DLS measurements. Shown are representative graphs out of four independent measurements. DLS, dynamic light scattering; EV, extracellular vesicles; HA, hyaluronic acid; HAS, hyaluronan synthase; Pi, inorganic phosphate; r1, T1-relaxivity of VSOP; r2, T2-relaxivity of VSOP; VSMC, vascular smooth muscle cells; VSOP, very small superparamagnetic iron oxide nanoparticles.

The knockdown of HAS1 and HAS2 using specific siRNA distinctly reduced the effects of Pi on the HA contents in cells and EV (Figure 3A). Only the knockdown of HAS2 significantly attenuated the HA levels induced by Pi. We subsequently investigated whether the knockdown of HAS2 in VSMC also affects the interaction of the cells with VSOP. Compared to control, Pi-treated cells strongly interacted with VSOP, indicated by high amounts of positively iron-stained areas after incubation of the cells with VSOP (Figure 3B). In contrast, a prior treatment of the cells with HAS2 siRNA reduced VSOP binding to the cells (Figure 3B). The HAS2 knockdown and the subsequently reduced HA levels also reduced the interaction of isolated EV with VSOP, as indicated by significantly reduced effects on the r1 and r2 relaxivity values of VSOP (Figure 3C). A knockdown of HAS1 in VSMC only slightly decreased the effects of isolated EV on the r1 and r2 relaxivities of VSOP (Figure 3C). In a parallel control experiment, we confirmed that treatment of the cells with HAS2-siRNA did not influence the levels of sulfated GAGs in cells and EV (Supplementary Figure S2). The decreased HA levels in EV also influenced their size. Dynamic light-scattering measurements revealed that EV from Pi-treated cells exhibited slightly larger hydrodynamic diameters compared to those of the control (Figure 3D). A previous treatment of the cells with HAS2 siRNA led to smaller hydrodynamic diameters induced by Pi (Figure 3D). EV derived from cells treated with HAS1 siRNA and Pi showed comparable hydrodynamic diameters to those of control EV (Figure 3D).

### 2.4. Inhibition of Wnt Signaling Blocks Pi-Induced Effects on HAS2 Expression and HA Contents in Cells and EV

**Figure 4:**
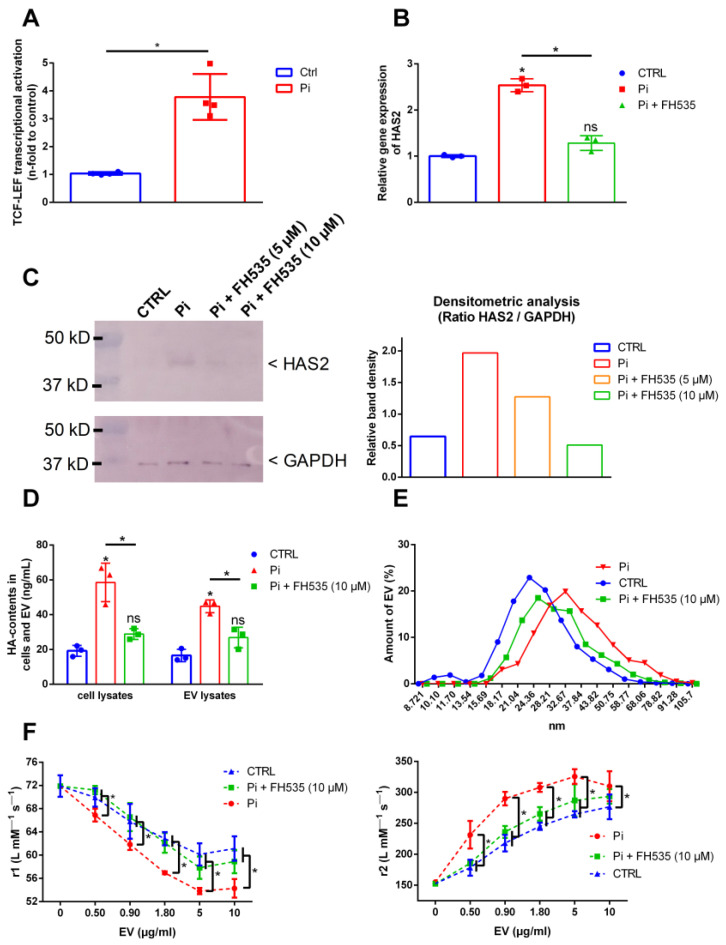
Inhibition of Wnt signaling blocks Pi-induced effects on HAS2 expression and HA contents in cells and EV. (**A**) VSMC were transfected with TCF/LEF-luciferase reporter plasmids and were treated with Pi for 3 d. Luciferase activity as an indicator for activation of Wnt signaling was measured. Data were normalized to a parallel transfected renilla reporter vector (means ± SD; n = 4; * *p* < 0.05). (**B**) Cells were treated with or without the presence of Pi or the Wnt antagonist FH535 for 3 d. HAS/2 protein expression was determined by Western blot (representative blots from three independent experiments). (**C**) VSMC were treated with Pi for 2 d with or without the presence of FH535. Gene expression of HAS2 was determined by qPCR and was normalized to RPL19 expression (means ± SD; n = 3; * *p* < 0.05 versus control and as indicated; ns, not significant). (**D**) VSMC were treated for 3 d with 3 mM Pi with or without the presence of FH535. Effects on HA contents in lysates of VSMC and VSMC-derived EV were analyzed by ELISA measurements (means ± SD; n = 3; * *p* < 0.05 compared to control and as indicated). (**E**) The effects of Wnt inhibition by FH535 on hydrodynamic diameters of EV from (**D**) were determined by DLS measurements (representative graphs out of three independent experiments). (**F**) Isolated EV from (**D**) were mixed at different concentrations with VSOP. Effects on VSOP relaxivities (r1, r2) were determined by linear fitting of T1- and T-2 relaxation rates in relation to VSOP concentrations (means ± SD; n = 4; * *p* < 0.05 as indicated). DLS, dynamic light scattering; EV, extracellular vesicles; FH535, Wnt antagonist; GAPDH, glyceraldehyde 3-phosphate dehydrogenase; HA, hyaluronic acid; HAS, hyaluronan synthase; Pi, inorganic phosphate; r1, T1-relaxivity of VSOP; r2, T2-relaxivity of VSOP; VSMC, vascular smooth muscle cells; VSOP, very small superparamagnetic iron oxide nanoparticles.

To gain further insights into the mode of action of Pi, we focused on the Wnt pathway, which is associated with a remodeling of the cardiovascular ECM during hyperphosphatemia. By applying a luminescent reporter gene assay, we found that Pi indeed induced the activation of Wnt signaling in VSMC (Figure 4A). Blocking the Wnt pathway using the Wnt antagonist FH535 led to reduced effects of Pi on the expression of HAS2 in VSMC on protein (Figure 4B) and gene expression levels (Figure 4C). FH535 also attenuated Pi-induced effects on HA levels in cells and isolated EV thereof (Figure 4D). Further, Wnt antagonism led to slightly reduced hydrodynamic diameters compared to Pi-induced EV without FH535 treatment (Figure 4E). In addition, the block of Wnt signaling led to significantly reduced effects of Pi-induced EV on the r1 and r2 relaxivity values of VSOP compared to EV induced by Pi without FH535 (Figure 4F).

### 2.5. Increased HA Contents in EV Increase Their Interaction with VSOP

**Figure 5:**
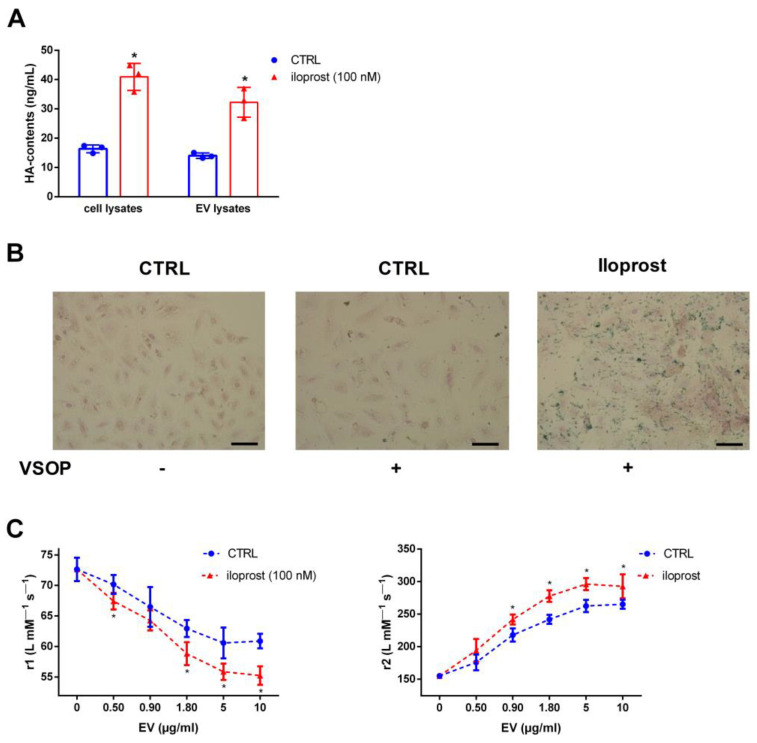
Increased HA contents in EV increase their interaction with VSOP. (**A**) VSMC were treated with or without the prostacyclin analogue iloprost for 3 d. Contents of HA in cell lysates and isolated EV from culture supernatants were determined by ELISA measurements (means ± SD; n = 3; * *p* < 0.05 compared to control). (**B**) Cells were treated as indicated for 3 d and were then incubated with VSOP. After washing of the cells, bound VSOP were visualized by iron staining and appeared blue. Representative images out of three independent experiments are shown. Bars = 50 µm. (**C**) Cells were treated with iloprost for 3 d prior to the isolation of EV from culture supernatants. Different EV concentrations were mixed with VSOP and effects on VSOP relaxivities (r1) were determined by linear fitting of T1-relaxation rates in relation to VSOP concentrations (means ± SD; n = 4; * *p* < 0.05 as indicated). EV, extracellular vesicles; HA, hyaluronic acid; r1, T1-relaxivity of VSOP; r2, T2-relaxivity of VSOP; VSMC, vascular smooth muscle cells; VSOP, very small superparamagnetic iron oxide nanoparticles.

Next, we investigated the relationship between HA contents and the interaction of cells and isolated EV with VSOP also independently of Pi. To carry this out, we applied the prostacyclin analogue iloprost to artificially increase the HA contents in VSMC. Figure 5A shows higher HA contents in iloprost-treated VSMC as well as in isolated EV thereof. Consecutive binding studies revealed stronger binding of VSOP to iloprost-treated cells compared to that observed in the control, indicated by higher amounts of positively iron-stained areas (Figure 5B). In addition, the effects of iloprost-induced EV on the decrease in r1 relaxivity values and on the increase in r2 relaxivity values of VSOP were significantly stronger compared to control EV (Figure 5C).

### 2.6. Exosome-Like EV Derived from Plasma of Rats with CKD Contain Elevated Levels of HA and Show a Strong Interaction with VSOP

**Figure 6:**
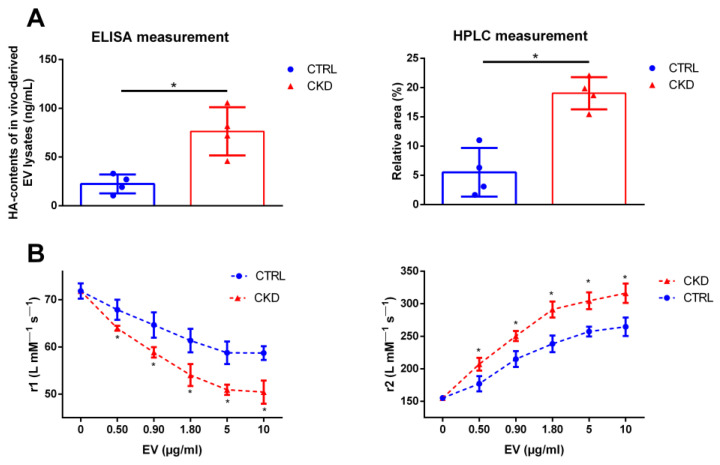
Exosome-like EV derived from plasma of rats with CKD contain elevated levels of HA and show a stronger interaction with VSOP compared to control. (**A**) HA contents in EV from plasma of rats with CKD and healthy control rats were determined by ELISA and HPLC measurements (means ± SD; n = 4; * *p* < 0.05). (**B**) Different concentrations of the plasma-derived EV were mixed with VSOP and effects on VSOP relaxivities (r1 and r2) were determined by linear fitting of T1- and T2-relaxation rates in relation to VSOP concentrations (means ± SD; n = 4; * *p* < 0.05 as indicated). CKD, chronic kidney disease; EV, extracellular vesicles; HA, hyaluronic acid; r1, T1-relaxivity of VSOP; r2, T2-relaxivity of VSOP; VSMC, vascular smooth muscle cells; VSOP, very small superparamagnetic iron oxide nanoparticles.

To check whether our in vitro-derived data are also of relevance for the in vivo situation, we finally analyzed the HA levels of in vivo-derived EV. CKD in rats was induced by adenine feeding and EV were isolated from the plasma. EV from the plasma of rats with CKD contained significantly more HA compared to EV from the plasma of healthy control rats, as shown by ELISA and HPLC measurements (Figure 6A). Similarly to Pi-induced EV from VSMC in vitro, the CKD-induced EV from rat plasma also showed significantly stronger effects on the r1 and r2 relaxivities of VSOP compared to control (Figure 6B).

## 3. Discussion

In this work, we provided evidence that the inorganic uremic toxin Pi increases the HA contents in VSMC and exosome-like EV isolated thereof. Higher HA contents lead to a stronger interaction of cells and EV with the imaging probe VSOP. Complementing previous data [5], this suggests the presence of negatively charged surface molecules like HA on cells and EV as potential targets for VSOP to visualize ECM-associated pathophysiological conditions by MRI.

We previously demonstrated a co-localization of EV in tissues rich in GAGs and VSOP [22]. In addition, we found that uremic conditions induce elevated contents of the sulfated GAGs heparan sulfate and chondroitin sulfate in vascular cells and in isolated exosome-like EV thereof. Higher contents of sulfated GAGs in turn promoted the interaction of the EV with VSOP [5]. However, this study focused on the GAGs heparan sulfate and chondroitin sulfate only rather than HA. HA contains only carboxylic groups, which are responsible for the negative charge of the molecule.

To further track down the functional role of negatively charged ECM molecules as targets for a visualization of pathologic tissue changes secondary to CKD, we here investigated the contribution of HA to the interaction between EV and VSOP. We initially found that treatment of VSMC with the uremic toxin Pi induces higher contents of HA in the cells and in isolated EV thereof. A first hint that higher HA contents impact the interaction between VSOP and cells and EV was deduced from cell binding studies and relaxometry measurements, respectively. The latter revealed a stronger effect of Pi-induced EV on the decline in T1-relaxivities of VSOP and on the increase in T2-relaxivities compared to the control. This indicates a strong interaction between both binding partners presumably via an increased agglomeration of VSOP cores around EV [22,23].

Further experiments revealed that the Pi-induced effects on HA in VSMC involved an upregulation of HAS1 and HAS2. These are two out of three known enzymes that exhibit hyaluronan synthase activity [11]. HAS catalyze the formation of HA chains by linking alternating glucuronic acid and N-acetylglucosamine residues via β-1,3 and β-1,4 glycosidic bonds.

The focus in the present study was on HAS2, which is primarily responsible for HA production in many cell types including VSMC and is distinctly more highly expressed than HAS3 [24,25]. In contrast to HAS1 and HAS2, HAS3 produces shorter HA chains, and its role seems to be mainly related to inflammation [26]. The effects of Pi and other uremic toxins on HAS3 will be investigated in follow-up studies including further cell types and tissue samples.

In accordance with higher HA levels, the upregulation of HAS, predominantly that of HAS2, contributes to the progression of cardiovascular diseases like atherosclerosis [15].

The expression of HAS is regulated by growth factors, cytokines, hormones, and transcription factors such as the platelet-derived growth factor or transforming growth factor-beta [27,28]. A novel aspect in our setting is the stimulatory effect of the uremic toxin Pi on the expression of HAS1 and HAS2 in VSMC.

A knockdown of both HAS led to reduced amounts of HA in cells and EV. Subsequent binding studies with VSOP revealed a decreased interaction with cells and EV due to decreased HA levels. The effects of a knockdown of HAS2 on HA levels in EV were stronger than those of HAS1. This might be due to the observation that HAS1-mediated synthesis of HA mainly occurs only under particular conditions, such as hyperglycemia [29].

Smaller hydrodynamic diameters of isolated EV reflected the lower HA levels compared to those of the control. EV with higher amounts of HA due to Pi treatment comprised larger hydrodynamic diameters. This is in line with other reports describing size-dependent modifications in exosomes with HA [30].

Several signaling pathways like the JNK/MAPK, TGF-β, and Wnt/β-catenin pathway can regulate the expression of HAS2 in cells [31,32]. The WNT/β-catenin pathway can be activated by Pi [33] and promotes the Pi-induced osteo-/chondrogenic transdifferentiation and calcification of VSMCs [34] and the remodeling of the cardiovascular ECM [35].

We therefore applied a reporter gene-based assay in VSMC to assess the effects of Pi on the activation of the T-cell factor/lymphoid enhancer factor (TCF/LEF) transcription factors. These function as major end point mediators of canonical Wnt signaling [36].

Pi treatment and subsequent upregulation of HAS2 went along with the activation of Wnt signaling. Vice versa, the inhibition of Wnt signaling blocked the Pi-induced expression of HAS2, thereby reducing the HA levels in cells and in isolated EV. This, in turn, diminished the interaction between VSOP and EV, which highlights Wnt signaling as a regulator of Pi-mediated effects on HA regulation in cells and EV.

Our hitherto results fit to the data from Golusda et al.’s work, who suggested HA is a main structure for VSOP binding and accumulation in a mouse model of colitis [20].

The exact mechanisms behind the interaction between HA and VSOP remain to be investigated in future studies. However, based on our relaxivity measurements, one can speculate about them.

The effects of EV on the relaxivities of VSOP can be referred to a process called transchelation. The negatively charged HA molecules on the surface of EV could function as substituents for the negatively charged citrate coating of positively charged iron oxide cores of VSOP. This interaction with EV—or HA molecules—might decrease the mobility of VSOP, thus affecting their local magnetic field. This, in turn, explains the diminished T1-relaxivity (r1) and increased T2-relaxivity (r2) values of VSOP in the presence of rising concentrations of EV [22,23]. This also fits to previous data on the usage of VSOP in the imaging of cells in T2-weighted MRI measurements [22].

To further prove that HA participates in the interaction between VSOP with cells and EV, we artificially increased the levels of HA in cells and EV using the prostacyclin analogue iloprost. A previous study demonstrated the stimulatory effects of iloprost on HAS2 expression in human VSMC with a subsequent increase in HA levels [37]. We observed similar effects in our study. Like Pi, treatment with iloprost increased the HA levels in cells and EV, which caused an augmented interaction between VSOP with cells and EV in binding and relaxivity measurements, respectively.

In accordance with a preceding study [5], the results from our cell culture experiments were reflected in analyses of plasma-derived EV from rats with CKD. The “CKD”-EV contained higher amounts of HA and showed a stronger interaction with VSOP compared to controls. In contrast to the in vitro-derived EV, the plasma-derived EV represent a mixture from different cellular sources such as endothelial cells or platelets [38]. This points to effects of Pi not only on the HA levels of VSMC but also of other cell types in the body, which complements other systemic effects of pathophysiological conditions in CKD [39].

Besides the interaction with VSOP, the Pi-induced alterations in HA levels could also be of relevance for other (patho)physiological processes. For instance, the accumulation of HA could promote proinflammatory actions such as an increased retention of macrophages [15]. Vice versa, decreased contents of HA are associated with a less pro-atherosclerotic phenotype of VSMC, which shows reduced migration and proliferation [40].

In addition to potential novel approaches for the design of molecular imaging probes, a modulation of HA contents is also of interest for therapeutic approaches using EV as a delivery system. The HA receptor cluster of differentiation (CD)44 could be overexpressed in specific tumor cells [41]. The preparation of HA-coated EV enabled Li et al. to increase the affinity of the EV to bind to the tumor cells and to increase the uptake of the cargo load of the EV by the tumor cells [30].

In conclusion, the results presented in this study might contribute to a better understanding of the pathophysiological processes in CKD. The uremic toxin Pi increases the amounts of negatively charged HA in cells and EV. Higher levels of HA, in turn, strengthen the interaction of cells and EV with VSOP. The findings might help the future development of novel EV- or tissue-selective imaging probes.

### Limitations

Our experiments were performed under static culture conditions. However, the flow conditions of the cell culture media can impact the cell surface, such as the structure of the glycocalyx [42]. Potential effects regarding the synthesis of HA need to be investigated in further studies. Nevertheless, a comparison between our in vitro- and in vivo-derived data points to the validity of our results.

A previous study reported possible contaminations of EV fractions with a high-molecular-weight HA fragment of 289 kDa, which could influence our measurements in this case [43]. However, the targeted up- and downregulation of HA in cells and EV by iloprost and siRNAs, respectively, and the according changes in the interaction of cells and EV with VSOP underline the validity of our conclusions.

## 4. Materials and Methods

### 4.1. Cell Culture

Rat aortic vascular smooth muscle cells (VSMC; A7r5 cell line, ATCC^®^ CRL-1444™) were cultured in DMEM (Gibco/ThermoFisher, Hennigsdorf, Germany) with 862 mg/L L-alanyl-L-glutamine, 1.0 g/L glucose, 50 μg/mL streptomycin, 50 units/mL penicillin and 10% heat-inactivated fetal bovine serum (FBS; Gibco). The cells were maintained at 70–80% confluence and were cultured in a humidified atmosphere at 37 °C and 5% CO_2_.

### 4.2. Treatment of Cells with Pi

VSMC were treated for 7 days in 75 cm^2^ or 125 cm^2^ flasks (NUNC, Roskilde, Denmark) with a standard culture medium containing 3.0 mM NaH_2_PO_4_ (Pi, final concentration) (Merck, Darmstadt, Germany). This concentration corresponds to values measured during chronic renal failure [44]. The medium was replaced every second day. Vehicle-treated cells served as the control.

For a later analysis of cells using qPCR, Western blot, the Blyscan^TM^-assay or ELISA, the cells were treated in 6-well or 12-well plates (NUNC).

### 4.3. Isolation and Characterization of EV

Following treatment with Pi, the cells were washed two times with sterile-filtered PBS and cultured for an additional 24 h in a standard culture medium supplemented with 5% exosome-depleted FBS (Gibco). The culture medium was then centrifuged at 700× *g* for 5 min to remove cells, followed by centrifugation at 2000× *g* for 20 min to remove cellular debris. The remaining supernatants were then concentrated using Vivaspin 20 centrifugal filter devices with 3K NMWL (GE Healthcare, Solingen, Germany) at 3220× *g* followed by centrifugation for 30 min at 20,000× *g* to obtain the microvesicle fraction. The supernatant was diluted 1:2 with the Total Exosome Isolation (from cell culture media) Reagent (Invitrogen/ThermoFisher) and incubated on a rotary shaker for 18 h at 4 °C. The exosome fraction was obtained by centrifugation of the solution for 60 min at 10,000× *g*. The pellet was washed twice with ice cold 0.9% NaCl solution (B. Braun Melsungen AG, Melsungen, Germany), resuspended in 100 µL ice cold 0.9% NaCl solution, and used immediately or stored for further analyses and experiments at −80 °C. The protein contents of the isolated EV fractions were used as a surrogate marker for the vesicle quantity. They were determined by the Pierce™ Rapid Gold BCA Protein-Assay-Kit (ThermoFisher).

EV from rat plasma were isolated with the Total Exosome Isolation (from plasma) Reagent (Invitrogen/ThermoFisher) without Proteinase K treatment. The pellets were washed, pooled, and stored in a NaCl solution as described above. The protein contents of the isolated EV fractions were determined as described above. Briefly, the EV samples (dissolved in NaCl) were diluted 1:4 with H_2_O and were directly subjected to the BCA assay according to the manufacturer’s instructions. The different EV populations of interest showed hydrodynamic diameters between 20 and 100 nm, as assessed by dynamic light-scattering measurements [5], and were positive for protein expression of the exosomal markers CD9, CD63 and CD81 [5].

### 4.4. Synthesis of VSOP

VSOP synthesis was described previously [45]. Briefly, iron (II) and iron (III) salts were dissolved in water and were precipitated as hydroxides at alkaline conditions. The addition of citric acid leads to the solubilization and stabilization of the iron oxide particles through the formation of a citrate coat.

### 4.5. T1- and T2-Relaxivity Measurements

The determination of proton T1-relaxation and T-2-relaxation rates were performed at 37 °C and 60 MHz (1.5 T) using a Minispec mq60 MR spectrometer (Bruker, Karlsruhe, Germany). The relaxation rates were determined with an inversion recovery pulse sequence, which is the standard measuring and analysis method of the equipment.

Measurements were performed in nanopure water containing 0.009% NaCl. T1-relaxivities of VSOP with and without the presence of EV were determined by mixing 0.0188 mM VSOP with different EV concentrations (protein contents between 0 and 10.0 µg/mL). Three solutions with different concentrations were measured for each sample. The relaxivity coefficients r1 and r2 were obtained by linear fitting of T1- and T2-relaxation rates and the values were normalized to the iron concentrations.

### 4.6. Determination of HA Contents in Cells and EV

The HA contents in cells and EV were determined using the HA Quantikine^TM^ ELISA (R&D Systems, Minneapolis, MN, USA). Cell culture supernatants were collected, centrifuged at 1000× *g* for 5 min to remove particulates, and stored at −20 °C. The remaining cells were washed with cold PBS and were lysed with the Cell Lysis buffer 2 (R&D Systems). After a centrifugation step at 1000× *g* for 5 min, the lysates were stored at −20 °C as well.

The ELISA measurements were performed according to the manufacturer’s instructions. Supernatants and lysates were applied in a 1:2 dilution and were analyzed in triplicate.

### 4.7. VSOP (Iron) Staining of Cells

After the incubation of the cells with VSOP, the cells were washed two times with water prior to incubation with Nuclear Fast Red Stain solution for 30 min (Roth, Karlruhe, Germany). After washing with water, the cells were treated with a solution consisting of a 1:1 mixture of 2% HCl and 2% potassium hexacyanoferrate (Merck). The cells were mounted with coverslips and were photographed within 15 min using an inverse DMi1 microscope equipped with a MC 120 HD microscope camera (Leica, Wetzlar, Germany).

### 4.8. Transfection of Cells with siRNA

By using the HiPerFect Transfection Reagent (Qiagen, Hilden, Germany), VSMC were transfected with siRNA specific for HAS1 or HAS2 (Silencer^®^, #AM16708, ThermoFisher) or a negative control (Silencer™ Negative Control No. 1 siRNA, #AM4611, ThermoFisher). Cells with ~75% confluence were treated with 2 µL siRNA and 10 µL transfection reagent in a 2 mL standard culture medium for 48 h, followed by 24 h incubation without siRNA and transfection reagent. The cells were then subjected to further analyses or were incubated for an additional 24 h with a standard culture medium containing 5% exosome-depleted FBS (ThermoFisher) to isolate EV from supernatants.

### 4.9. Western Blot Analyses

Treated cells or pellets of isolated EV were lysed with 100 μL lysis buffer and protein concentrations were determined using the Pierce™ Rapid Gold BCA Protein-Assay-Kit (ThermoFisher). Equivalent amounts of protein were subjected to 10% Mini-PROTEAN^®^ TGX™ Precast Protein Gels (Bio-Rad, Feldkirchen, Germany). Gels were blotted using a Mini Trans-Blot^®^ Cell (Bio-Rad) onto Amersham™ nitrocellulose membranes (Merck). All primary antibodies used are given in Table 1. Bound antibodies on the membranes were visualized using anti-mouse or anti-rabbit WesternBreeze^®^ Chromogenic Immunodetection System Kits (ThermoFisher). Band densities were quantified using Image J software (version 1.50i; National Institutes of Health, Bethesda, MA, USA).

### 4.10. Analysis of Wnt Signaling in VSMC

Effects of Pi on Wnt signaling in VSMC were analyzed using a luminescent reporter plasmid assay. Briefly, VSMC were transiently transfected with reporter plasmids (Promega, Mannheim, Germany) for the T-cell factor (TCF)/lymphoid enhancer-binding factor (LEF) binding motif (pGL4.49[luc2P/TCF-LEF RE/Hygro]) and a renilla vector (pGL4.74[hRluc/TK]). To inhibit Wnt activation, the β-Catenin/TCF site-inhibitor FH535 (10 µM; Merck) was used.

### 4.11. Manipulation of HA Levels in Cells

To artificially increase the HA contents, the cells were treated with 100 nM iloprost (Sigma) for 72 h. Afterwards, the cells were incubated for 24 h with a standard culture medium containing 5% exosome-depleted FBS (ThermoFisher) to isolate EV from supernatants, as described above.

### 4.12. HA Analysis of EV by HPLC

The quantification of HA was performed by high-performance liquid chromatography (HPLC) as described in [46]. Briefly, EV pellets were proteolytically digested and proteoglycans were cleaved into disaccharides, labeled with 2-animobenzamide and analyzed by HPLC equipped with fluorescence detection.

### 4.13. PCR Measurements

The relative gene expressions in cells were determined by qPCR using TaqMan™ assays. A list of all Taqman probes applied is given in Table 2. The gene expressions in samples relative to the controls’ were determined by using the 2^−∆∆Ct^ method and normalized to the gene expression of the housekeeping gene *ribosomal protein L19* (*RPL19*).

### 4.14. Statistics

Statistics were calculated using GraphPad Prism (GraphPad Software, Version. 6.01, La Jolla, CA, USA). The data sets were tested for outliers and normal distribution. The in vitro data were analyzed as follows: two treatment groups were compared by using unpaired *t*-tests. Two treatment groups with different variables were compared by 2-way ANOVA and Sidak’s multiple comparisons test. Comparisons of three groups were carried out by 1-way ANOVA and Tukey’s multiple comparisons test. The in vivo data were analyzed by the Mann–Whitney test. *p*-values of < 0.05 were considered statistically significant.

## Figures and Tables

**Table 1:** Primary antibodies used for Western blot analyses.

Target	Dilution	Company	Product Number
HAS1	1:800	ThermoFisher, Hennigsdorf, Germany	#PA595599
HAS2	1:800	ThermoFisher	#PA5115388
GAPDH	1:1000	ThermoFisher	#A5-15738

**Table 2:** List of applied Taqman probes (ThermoFisher) in the gene expression experiments.

Gene	Full Name	Assay-ID (Rat)
*HAS1*	*Hyaluronan synthase 1*	Rn01455687_g1
*HAS2*	*Hyaluronan synthase 2*	Rn00565774_m1
*RPL19*	*Ribosomal Protein L19*	Rn00821265_g1

## Data Availability

The data that support the findings of this study are available from the corresponding author upon reasonable request.

## Supplementary Materials

The supporting information can be downloaded at: https://www.mdpi.com/article/10.3390/ijms25052571/s1.

## Abbreviations

CKD: chronic kidney disease; EV, extracellular vesicles; FH535, synthetic Wnt pathway inhibitor; GAG, sulfated glycosaminoglycans; Pi, inorganic phosphate; r1/r2, T1/T2-relaxivities: properties of a contrast agent like VSOP, which are affected by the interaction of VSOP with surrounding binding partners; HPLC, high-performance liquid chromatography; VSMC, vascular smooth muscle cells; VSOP, very small superparamagnetic iron oxide nanoparticles—a contrast agent for magnetic resonance imaging measurements.
