# Supplementary material for: Inorganic Phosphate-Induced Extracellular Vesicles from Vascular Smooth Muscle Cells Contain Elevated Levels of Hyaluronic Acid, Which Enhance Their Interaction with Very Small Superparamagnetic Iron Oxide Particles
Source: Int J Mol Sci. 2024 Feb 22;25(5):2571. doi: 10.3390/ijms25052571 (PMC10932207; doi:10.3390/ijms25052571)
Supplement: Supplementary file 1 [file ijms-25-02571-s001.zip › Supplemental Figure S1.pdf]

Supplemental Figure S1.

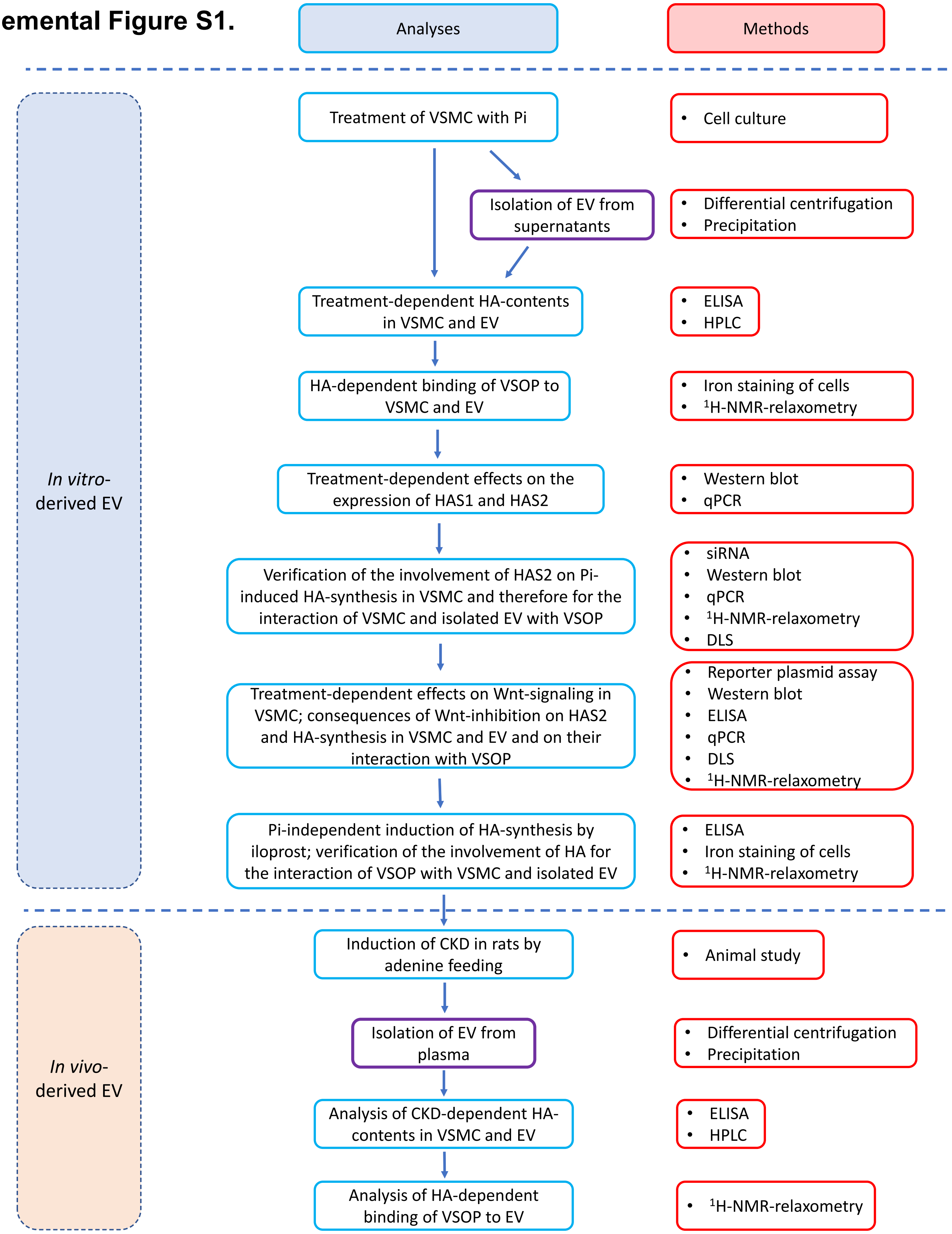

Supplemental Figure S1. Diagram of the research process. Initially, *in vitro* studies using rat vascular smooth muscle cells and isolated EV thereof were performed. To validate these results, EV were isolated from rats with CKD and healthy control rats and similar analyses regarding HA-contents in EV and the interaction of the EV with VSOP were performed. **Abbreviations**: *CKD, chronic kidney disease; DLS, dynamic light scattering; EV, exosome-like extracellular vesicles; ^{1}H-NMR-relaxometry, method to determine magnetic properties of contrast agents like VSOP; HA, hyaluronic acid; HAS, hyaluronic acid synthase; Pi, inorganic phosphate; VSMC, vascular smooth muscle cells; VSOP, very small superparamagnetic iron oxide nanoparticles.*
