# Supplementary material for: Inorganic Phosphate-Induced Extracellular Vesicles from Vascular Smooth Muscle Cells Contain Elevated Levels of Hyaluronic Acid, Which Enhance Their Interaction with Very Small Superparamagnetic Iron Oxide Particles
Source: Int J Mol Sci. 2024 Feb 22;25(5):2571. doi: 10.3390/ijms25052571 (PMC10932207; doi:10.3390/ijms25052571)
Supplement: Supplementary file 1 [file ijms-25-02571-s001.zip › Supplemental Figure S2.pdf]

Supplemental Figure S2.

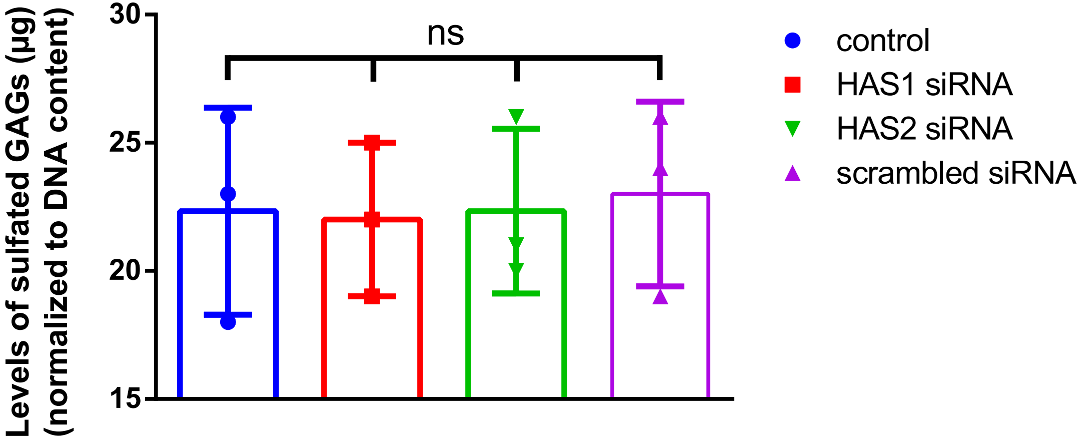

Supplemental Figure S2.
Effects of HAS1 and HAS2 specific siRNA on the contents of sulfated GAGs in VSMC. VSMC were transfected with siRNA specific for HAS1, HAS1 or a scrambled control siRNA (Silencer®, Thermo-fisher) for 48 h by using the HiPerFect Transfection Reagent (Qiagen, Hilden, Germany). This was followed by an additional incubation of 24 h without siRNA and transfection reagent. Cells were then directly subjected to respective analyses. To isolate EV, the cells were incubated for additional 24 h with standard culture medium containing 5% exosome-depleted FBS (ThermoFisher) and EV were isolated from supernatants as described [1]. GAG contents in cells and isolated EVs were determined using the Blyscan^{TM} assay as described [1]. Shown are means ± SD from three independent experiments.

[1] Freise C., Zappe A., Löwa N., (…), Wiekhorst F. and Taupitz M. Uremic toxin-induced exosome-like extracellular vesicles contain enhanced levels of sulfated glycosaminoglycans which facilitate the interaction with very small superparamagnetic iron oxide particles. Int J Mol Sci, 2023; 24, 14253. doi: 10.3390/ijms241814253
